# Dietary folate drives methionine metabolism to promote cancer development by stabilizing MAT IIA

**DOI:** 10.1038/s41392-022-01017-8

**Published:** 2022-06-22

**Authors:** Jin-Tao Li, Hai Yang, Ming-Zhu Lei, Wei-Ping Zhu, Ying Su, Kai-Yue Li, Wen-Ying Zhu, Jian Wang, Lei Zhang, Jia Qu, Lei Lv, Hao-Jie Lu, Zheng-Jun Chen, Lu Wang, Miao Yin, Qun-Ying Lei

**Affiliations:** 1 Fudan University Shanghai Cancer Center & Institutes of Biomedical Sciences; Cancer Institutes; Key Laboratory of Breast Cancer in Shanghai; Shanghai Key Laboratory of Radiation Oncology, the Shanghai Key Laboratory of Medical Epigenetics, Department of Oncology, Shanghai Medical College, Fudan University, Shanghai, 200032 People’s Republic of China; 2 Department of Hepatic Surgery, Fudan University Shanghai Cancer Center, Shanghai Medical College, Fudan University, Shanghai, 200032 People’s Republic of China; 3 MOE Key Laboratory of Metabolism and Molecular Medicine, Department of Biochemistry and Molecular Biology, School of Basic Medical Sciences, Fudan University, Shanghai, 200032 People’s Republic of China; 4 State Key Laboratory of Cell Biology, Shanghai Institute of Biochemistry and Cell Biology, Center for Excellence in Molecular Cell Science, Chinese Academy of Sciences (CAS), School of Life Science and Technology, Shanghai Tech University, Shanghai, 200032 People’s Republic of China; 5 State Key Laboratory of Medical Neurobiology, Fudan University, Shanghai, 200032 People’s Republic of China

**Keywords:** Cancer metabolism, Gastrointestinal cancer

## Abstract

Folic acid, served as dietary supplement, is closely linked to one-carbon metabolism and methionine metabolism. Previous clinical evidence indicated that folic acid supplementation displays dual effect on cancer development, promoting or suppressing tumor formation and progression. However, the underlying mechanism remains to be uncovered. Here, we report that high-folate diet significantly promotes cancer development in mice with hepatocellular carcinoma (HCC) induced by DEN/high-fat diet (HFD), simultaneously with increased expression of methionine adenosyltransferase 2A (gene name, MAT2A; protein name, MATIIα), the key enzyme in methionine metabolism, and acceleration of methionine cycle in cancer tissues. In contrast, folate-free diet reduces MATIIα expression and impedes HFD-induced HCC development. Notably, methionine metabolism is dynamically reprogrammed with valosin-containing protein p97/p47 complex-interacting protein (VCIP135) which functions as a deubiquitylating enzyme to bind and stabilize MATIIα in response to folic acid signal. Consistently, upregulation of MATIIα expression is positively correlated with increased VCIP135 protein level in human HCC tissues compared to adjacent tissues. Furthermore, liver-specific knockout of *Mat2a* remarkably abolishes the advocating effect of folic acid on HFD-induced HCC, demonstrating that the effect of high or free folate-diet on HFD-induced HCC relies on *Mat2a*. Moreover, folate and multiple intermediate metabolites in one-carbon metabolism are significantly decreased in vivo and in vitro upon *Mat2a* deletion. Together, folate promotes the integration of methionine and one-carbon metabolism, contributing to HCC development *via* hijacking MATIIα metabolic pathway. This study provides insight into folate-promoted cancer development, strongly recommending the tailor-made folate supplement guideline for both sub-healthy populations and patients with cancer expressing high level of MATIIα expression.

## Introduction

Methionine metabolic cycle, coupled with folate cycle, contributes to one-carbon units (methyl groups) metabolism. In mammals, folate and methionine were converted to reduced tetrahydrofolate (THF) and S-adenosylmethionine (SAM), respectively, which are involved in ^1^C unit cycle to support various essential cellular events, such as purine and thymidine biosynthesis, anti-redox condition, epigenetic regulation, and amino acid homeostasis.^1,2^

Methionine implicates in cancer development through enhancing the biosynthesis of protein and nucleotide and/or epigenetically regulating genome-wide methylation. SAM, the major methyl group donor in cells, is generated by methionine adenosyl transferases (MATs)-catalyzed enzymatic process. Among three MAT iso-enzymes, MAT I and III are polymers constituted of α1 catalytic subunit encoded by *MAT1A* gene, whereas α2 catalytic subunit is the product of *MAT2A* gene and form MAT II with *MAT2B*-encoded β regulatory subunit.^3,4^ *MAT1A* and *MAT2A* expression are under temporal and spatial control.^5^ For example, MAT1A is mainly detected in adult liver. By contrast, MATIIα is present in embryonic liver and other tissues. In addition to transcriptional regulation, post-translational modification (PTM) of MATIIα has been discovered.^6,7^ Acetylation at MATIIα lysine residue reduces its protein stability through the ubiquitin-proteasome pathway. The upregulation of *MAT2A* gene expression has been found in cancers.^8–10^ Moreover, lung tumor-initiating cells (TICs) are predisposed to MATIIα-promoting methionine metabolism using the xenograft mouse model. A very recent study revealed that diet with reduced methionine could effectively ameliorate cancer outcomes, accompanied by the systemic decrease of methionine-related metabolites.^11,12^

In our present study, we found that dietary folate promotes tumor development in DEN/High-fat diet (HFD)-induced hepatocellular carcinoma (HCC) mouse model along with increased MATIIα protein level and acceleration of methionine cycle. Consistently, high expression of *MAT2A* indicates poor prognosis in different human cancers. Mechanistically, we identified VCIP135 as a deubiquitylating enzyme to prevent MATIIα degradation from the ubiquitin-proteasome pathway in response to folic acid deficiency. Furthermore, liver-specific *Mat2a* knockout significantly impairs HCC tumorigenesis. Notably, the effect of high or free folate-diet on DEN/HFD-induced HCC relies on *Mat2a*. Moreover, RNA-seq analysis revealed that *Mat2a* loss causes dysregulation of genes involved in cell proliferation and metastasis. Additionally, the functional assay demonstrated that double knockdown *MAT2A* and/or *VCIP135* significantly represses cancer cell growth. More importantly, folate and other intermediate metabolites, like SAM and SAH, in one-carbon metabolism are remarkably decreased in vivo and in vitro when silencing *MAT2A*. Additionally, we defined the positive correlation between the expression level of VCIP135 and MATIIα in HCC and the enrichment of one-carbon metabolites in cancer tissues compared with that in adjacent tissues.

## Results

### MATIIα positively correlates with folic acid-promoted HCC development

**Fig. 1:**
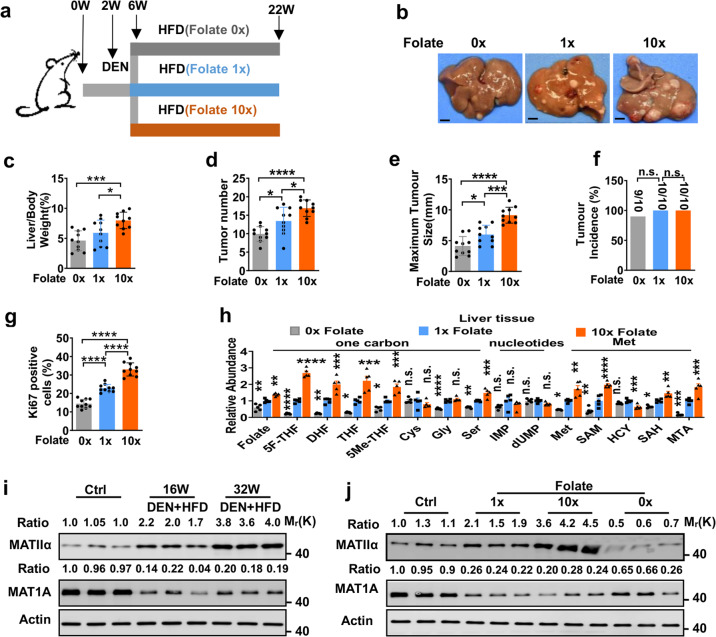
MATIIα positively correlates with folic acid-promoted HCC development. **a** Schematic representation of DEN/HFD-induced HCC model. **b** Representative images from 22-week-old DEN/HFD-treated mice fed with different concentration folate diet as indicated. **c**–**e** Quantification of the ratio of liver weight to body weight (**c**), the number of tumors (**d**), and the size of the largest tumor per mouse (**e**). Mean ± s.e.m. of *n* = 10 biologically independent experiments, one-way ANOVA test. **f** Quantification of tumor incidence in mice fed with different folate acid diet. Mean ± s.e.m., one-way ANOVA test. **g** Quantification of Ki67 IHC staining of mice liver tissues. Ten fields each, mean ± s.e.m., one-way ANOVA test. **h** Quantification of different metabolites of liver tissue in mice fed with different folate diets. Mean ± s.e.m. of *n* = 5 biologically independent experiments, one-way ANOVA test. **i**, **j** Immunoblots of MatIIα and Mat1A in liver tissues from indicated mice. WT mice was employed as Ctrl (control) mice. Data in **i** and **j** are representative of three independent experiments. n.s. donates for no significance, *P < 0.05, **P < 0.01, ***P < 0.001 and ****P < 0.0001

Folate is essential for rapid cell proliferation and plays a pivotal role in one-carbon metabolism. But the role of folate and its synthetic form, folic acid, in cancer development and progression is highly controversial.^7,13–16^ To explore how folate influence the development of HCC in vivo, we fed DEN-injected mice with purified HFD containing standard level of folate (1×), high level of folate (10×) or folate free (0×) as indicated, respectively, because diet is the main source of folate in the body (Fig. 1a). 5 months after DEN injection, high folate diet significantly promoted HFD-induced HCC development (Fig. 1b). Quantitative analyses revealed that high folate diet significantly increased the ratio of liver/body weight, the total number of visible tumors and maximum tumor size in liver (Fig. 1c–e). In contrast, folate free diet markedly blunted HFD-induced HCC development (Fig. 1b). The ratio of liver/body weight of DEN-injected mice fed with folate-free diets were significantly decreased, along with a significantly reduced total number of visible tumors and maximum tumor size in liver (Fig. 1c–e). At the same time, we found no significant difference of tumor incidence, food intake, and body weight among these three groups (Fig.1f and Supplementary Fig. S1a, b). Next, Ki67 immunostaining analysis showed that cell proliferation was significantly enhanced in high folate group or inhibited in folate free group, compared with that in standard level of folate-fed mice group, respectively (Fig. 1g and Supplementary Fig. S1c). Furthermore, we tested the intermediates concentration of folate and methionine cycle in serum and liver of mice fed with diet containing different folate contents. In serum, high folate diet increased while folate free diet decreased the intermediates levels of folate metabolism. Neither high nor folate-free diet had an effect on the intermediate levels of methionine cycle (Supplementary Fig. S1d). In liver tissue, high folate diet increased while folate free folate diet decreased the intermediates levels of folate metabolism and methionine cycle. Neither high folate nor folate-free diet had an effect on levels of IMP and dUMP, the intermediates of nucleotides synthesis (Fig. 1h). As amount of folate intake critically regulates HCC development, it is reasonable that methionine level is altered in response to diets containing different concentrations of folate. Homocysteine (Hcy) is the intermediate metabolite of methionine cycle and can be remethylated to generate methionine. Increased Hcy level is linked to cancer.^17^ However, it has been demonstrated that nutrition intervention supplemented with folate or 5-MTHF effectively downregulates serum level of Hcy in multiple clinical trials.^18–20^ It may result in unchanged level of Hcy as mice were fed with folate-enriched diets in our study. According to data shown in Fig. 1h, levels of intermediate metabolites of folate metabolism, like 5F-THF, DHF, THF, and 5Me-THF, are significantly affected by the amount of folate intake in our study. Moreover, we performed H&E staining to analyze the effects of folate starvation on different organs from mice fed with folate-free or normal diet. No significant histological differences were observed in heart, spleen, kidney, pancreas, and muscle (Supplementary Fig. S1e). However, among 5 of 7 mice fed with folate free diet, pulmonary vascular amyloid lesions in lung were observed (Supplementary Fig. S1e). Liver carcinogenesis is often associated with liver injury.^21^ As expected, we found that serum levels of alanine transaminase (ALT) and aspartate transaminase (AST) were markedly elevated in high folate-fed mice but decreased in folate free-fed mice compared with normal level of folate-fed mice, respectively (Supplementary Fig. S1f, g). We then detected MATIIα protein level because of the close linkage between methionine cycle and folate transition. It showed that MATIIα protein level was upregulated in HCC tissues from mice with 16-week DEN/HFD administration and higher protein level of MATIIα was observed in HCC tissues from mice with 32-week DEN/HFD administration (Fig. 1i). In contrast, MAT1A protein level was dramatically downregulated in mice HCC tissues with 16- or 32-week DEN/HFD treatment (Fig. 1i). Notably, DEN/HFD administration-upregulated MATIIα protein level was further enhanced by the addition of high folate diet and largely diminished by feeding with folate free diet even in presence of DEN/HFD, while MAT1A protein level had inverse expression (Fig. 1j). In agreement with previous findings,^6,22–24^ we also found that the survival period of patients with different cancers displaying high *MAT2A* expression level became significantly shorter by analyzing overall survival (Supplementary Fig. S1h). High expression level of *MAT2A* in liver cancer, breast cancer, cervical squamous cell carcinoma, and gastric cancer was significantly associated with poor outcomes (Supplementary Fig. S1h). Taken together, these results suggest that dietary folate promotes HCC development, simultaneously increasing MATIIα expression.

### DUB VCIP135 stabilizes MATIIα to promote cell proliferation by sensing folic acid

**Fig. 2:**
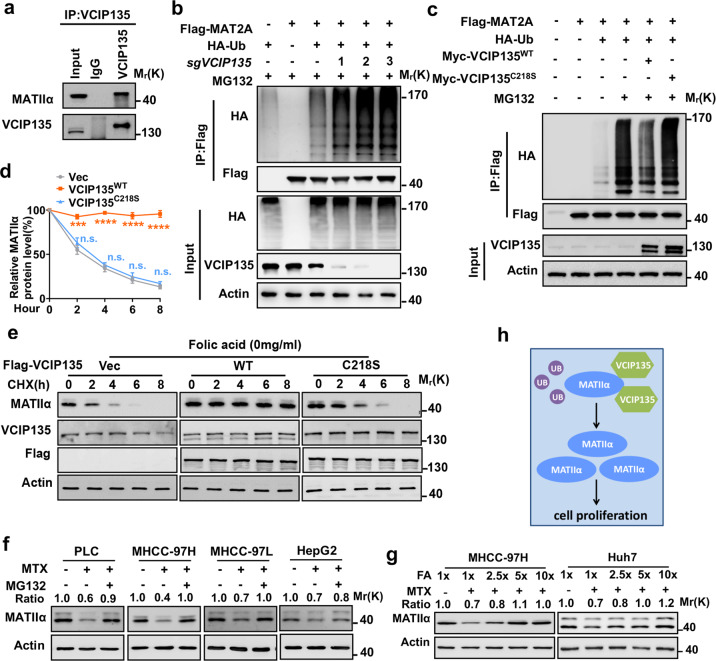
DUB VCIP135 stabilizes MATIIα to promote cell proliferation by sensesing folic acid. **a** VCIP135 endogenously interacts with MATIIα. **b** *VCIP135* knockdown increases MATIIα ubiquitylation. HEK293T cells were transfected with the indicated plasmids. **c** VCIP135 WT but not enzymatic-dead mutant C218S decreases MATIIα ubiquitylation. HEK293T cells were transfected with the indicated plasmids. **d**, **e** VCIP135 WT but not enzymatic-dead mutant stabilizes MATIIα upon folate deprivation for 72 h. MHCC-97H cells were cultured under folate-deprived condition, followed by CHX treatment. Mean ± s.d. of *n* = 3 (**d**) biologically independent experiments, one-way ANOVA test. **f** MG132 rescues MTX-reduced MATIIα protein level in multiple HCC cell lines. **g** Folate rescues MTX-reduced MATIIα protein level in MHCC-97H and Huh7 cells. **h** Working model. VCIP135 deubiquitylates MATIIα in response to folate and induces MATIIα accumulation in protein level. Data (**a**–**c** and **e**–**g**) are representative of three independent experiments. n.s. donates for no significance, ***P < 0.001 and ****P < 0.0001

Our previous studies have demonstrated that MATIIα stability was regulated by E3 ligase UBR4.^7^ Next, we used tandem affinity purification and mass spectrometry (TAP-MS) to identify the potential deubiquitylase of MATIIα in response to folate signal. The MS analysis found the candidates of MATIIα-interacted proteins such as MTR, RNF138, and VCIP135, as listed in Supplementary Fig. S2a. We then explored whether VCIP135 acts as deubiquitylase of MATIIα because it was the only identified deubiquitylase among the MATIIα-interactors. VCIP135 possesses deubiquitylating activity, which is essential for p97/p47-mediated Golgi membrane fusion.^25^ We thus speculated that VCIP135 could regulate MATIIα protein stability through direct interaction and deubiquitylating MATIIα. To this end, endogenous VCIP135 was immunoprecipitated and subjected to immunoblot, MATIIα was readily detected in precipitates (Fig. 2a). We then found that UBR4-reduced MATIIα protein level was dramatically restored by VCIP135 overexpression (Supplementary Fig. S2b). Furthermore, knockdown of *VCIP135* showed enhanced MATIIα ubiquitylation (Fig. 2b). On the contrary, immunoblotting results showed that the MATIIα ubiquitylation was substantially inhibited by VCIP135 WT but not enzymatic-dead C218S mutant (Fig. 2c). Consistently, the CHX chase experiment showed that knockdown of *VCIP135* significantly shortened the half-life of MATIIα (Supplementary Fig. S2c, d). Our previous studies have indicated that folate affected the stability of MATIIα in HCC and CRC cell lines.^7,26^ We further found that VCIP135 could extend the half-life of MATIIα and rescue the ubiquitylated degradation of MATIIα upon folate deprivation (Fig. 2d, e and Supplementary Fig. S2e). Moreover, we observed that folate treatment did not affect the binding between VCIP135 and MATIIα and the activity of VCIP135^27^ (Supplementary Fig. S2f, g) while folate deprivation downregulated MATIIα and VCIP135 protein levels in multiple tumor cell lines (Supplementary Fig. S2h).

The chemotherapeutic drug methotrexate (MTX) inhibits the enzyme DHFR (dihydrofolate reductase) which generates tetrahydrofolate (THF) from dihydrofolate, disturbing folate/one carbon cycle. Here, we found that MTX inhibits MATIIα protein level. Notably, the inhibitory effect of MTX was rescued by MG132 treatment to different degrees in the tested HCC cell lines (Fig. 2f). Furthermore, we found that MTX-decreased MATIIα protein level was rescued by excess folate in a dose-dependent manner in both MHCC-97H cells and Huh-7 cells (Fig. 2g). Collectively, these results demonstrate that VCIP135 functions as a DUB targeting MATIIα to prevent the proteasomal degradation pathway in response to folate (Fig. 2h). Together, DUB VCIP135 senses folic acid to stabilize MATIIα for promoting cell proliferation.

### MATIIα is crucial for high fat-induced HCC progression

**Fig. 3:**
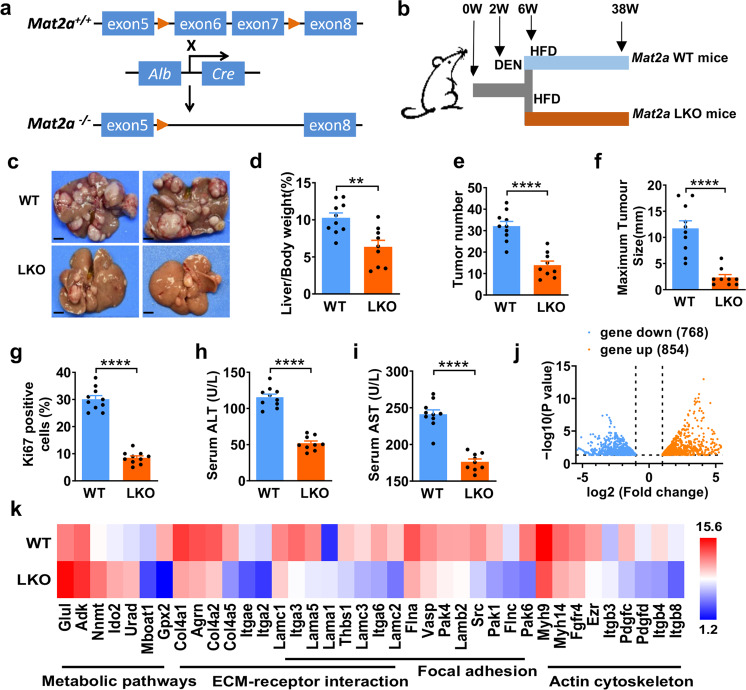
MATIIα is crucial for high fat-induced liver cancer progression. **a** Generation of liver tissue-specific *Mat2a*-knockout mice. LoxP was indicated as arrowhead. **b** Schematic representation of DEN/HFD-induced HCC model. **c** Representative images of mouse livers from the indicated group. *n* = 10 (WT), *n* = 9 (LKO) biologically independent experiments. Scale bar, 5 mm. Quantification of the ratio of liver weight to body weight (**d**) and the number of tumors (**e**), quantification of size of the largest tumor per mouse (**f**). Mean ± s.e.m. of *n* = 10 (WT), *n* = 9 (LKO) biologically independent experiments, two-tailed t-test. **g** Quantification of Ki67 staining of mice liver tissues. Ten fields each, mean ± s.e.m., two-tailed t-test. **h**, **i** ALT and AST contents in mice serum. Mean ± s.e.m. of *n* = 0 (WT), *n* = 9 (LKO) biologically independent experiments, two-tailed t-test. **j** Volcano map shows the altered genes and pathways in mice liver *via* RNA-seq after *Mat2a* specific knockout in the hepatocytes with 16-week DEN/HFD treatment. **k** Gene expression levels of the subsets of genes with significant changes in the metabolic pathways, ECM-receptor interaction, Focal adhesion, Actin cytoskeleton in LKO mice at 16 weeks. *n* = 4 in each group. **P < 0.01, and ****P < 0.0001

Given increased MATIIα expression in liver tissue of HFD-fed mice bearing HCC, we employed the liver-specific *Mat2a* knockout (LKO) mouse model (Fig. 3a and Supplementary Fig. S3a) to address the contribution of MATIIα to the promotion of HCC development during over-nutrition. In this case, mice with HFD feeding for 32 weeks were followed up (Fig. 3b), we observed that *Mat2a* LKO dramatically blunted HFD-induced HCC development compared to *Mat2a* wild-type (WT) mice (Fig. 3c). Quantitative analyses revealed that HFD-fed *Mat2a* LKO mice had significant decrease of the ratio of liver/body weight (Fig. 3d), as well as remarkable reduction of visible tumor numbers and maximum tumor size (Fig. 3e, f). It was notable that there was no difference of tumor incidence in these two groups (Supplementary Fig. S3b). Moreover, cell proliferation was profoundly suppressed in *Mat2a* LKO mice compared with WT controls by analyzing Ki-67 staining (Fig. 3g and Supplementary Fig. S3c). Consistently, we detected significantly lower serum levels of ALT and AST in *Mat2a*-LKO mice than those in the *Mat2a* WT mice (Fig. 3h, i). Non-alcoholic steatohepatitis (NASH) is the most severe form of non-alcoholic fatty liver disease (NAFLD) and supposed as a potential precursor of HCC.^28^ 16 weeks after HFD feeding, fasting serum glucose and insulin levels were significantly decreased in *Mat2a* LKO mice (Supplementary Fig. S3d, e). Furthermore, glucose and insulin tolerance tests (GTT and ITT, respectively) revealed that *Mat2a* deletion protected against HFD-induced glucose intolerance and insulin resistance (Supplementary Fig. S3f, g). Furthermore, we performed targeted metabolite profiling and found that Met was increased while SAM was decreased in both in *Mat2a*-KO adjacent tissue and HCC tissue (Fig. S3h) as the conversion of Met to SAM was disrupted by *Mat2a* KO. Further analysis revealed that the level of SAM and SAH showed reduction in LKO-HCC compared to control-HCC. Interestingly, folate (FA), SAM, SAH, Hcy, and Gly displayed no variation in adjacent tissues by comparing control and LKO groups (Fig. S3h). To dissect the mechanism of MATIIα in HCC development, we conducted gene expression profile analysis using WT and *Mat2a*-deficient liver tissues. RNA-seq assay revealed that genes with expression change in adjacent normal liver tissues could be categorized into three groups including metabolic pathways, ECM-receptor interaction, and focal adhesion by comparing Mat2a-KO and control mice (Fig. S4a, b). Following 16 weeks HFD feeding, genes with distinct expression changes in HCC between *Mat2a* WT and KO groups were classed into four categories, including (i) Metabolic pathways, (ii) ECM-receptor interaction, (iii) Focal adhesion, and (iv) Regulation of actin cytoskeleton (Fig. 3j, k and Supplementary Fig. S4c). The altered gene expression was further validated by qPCR (Supplementary Fig. S4d–g). Particularly, we chose the candidate genes involved in cell proliferation (NNMT) and metastasis (PAK1) to perform functional assay, confirming our findings. Consistent with previous reports,^29,30^ we found that *NNMT* knockdown enhanced cell proliferation in MHCC-97H cells (Supplementary Fig. S4h) and *PAK* knockdown decreased cell migration in MHCC-97H cells (Supplementary Fig. S4i, j). Collectively, MATIIα is critical for high fat-induced HCC progression.

### MATIIα is essential for folic acid-promoted HCC progression

**Fig. 4:**
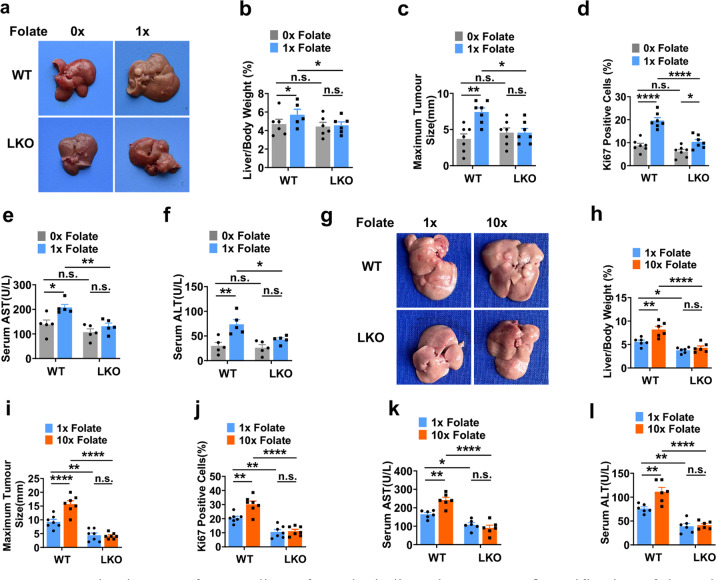
MATIIα is essential for folic acid-promoted cancer development. **a** Representative images of mouse livers from the indicated group. **b**, **c** Quantification of the ratio of liver weight to body weight (**b**) and the size of the largest tumor per mouse (**c**) fed with folate free diet. Mean ± s.e.m. of *n* = 5–7 biologically independent experiments, one-way ANOVA test. **d** Quantification of Ki67 staining of mice liver tissues. Seven fields each, mean ± s.e.m., one-way ANOVA test. **e**, **f** Serum contents of AST (**e**) and ALT (**f**) contents in mice that fed with indicated folate diet. Mean ± s.e.m. of *n* = 5 biologically independent experiments, one-way ANOVA test. **g** Representative images of mouse livers from the indicated group. **h**, **i** Quantification of the ratio of liver weight to body weight (**h**) and the size of the largest tumor per mouse (**i**) fed with folate free diet. Mean ± s.e.m. of *n* = 5–7 biologically independent experiments, one-way ANOVA test. **j** Quantification of Ki67 staining of mice liver tissues. Seven fields each, mean ± s.e.m., one-way ANOVA test. **k**, **l** Serum contents of AST (**k**) and ALT (**l**) contents in mice that fed with indicated folate diet. Mean ± s.e.m. of *n* = 6 biologically independent experiments, one-way ANOVA test. n.s. donates for no significance, *P < 0.05, **P < 0.01, and ****P < 0.0001

We then investigated whether HCC development promoted by dietary folate is depended on MATIIα. We fed HFD-mice with free or normal folate diet for 16 weeks then harvested (Supplementary Fig. S5a). Folate free diet indeed dramatically attenuated HFD-induced HCC development in *Mat2a* WT but not KO mice (Fig. 4a). Quantitative analyses revealed that folate free-feeding significantly reduced the ratio of liver/body weight and maximum tumor size in liver in *Mat2a* WT but not KO mice (Fig. 4b, c). Furthermore, Ki67 staining demonstrated that folate free diet resulted in significant decrease of cell proliferation in both *Mat2a* WT and KO mice while suppressive effect of cell proliferation in *Mat2a* WT mice was more dramatic than that of *Mat2a* KO mice (Fig. 4d and Supplementary Fig. S5b). As expected, the serum levels of AST and ALT were markedly decreased in HFD-fed mice coupled with folate free diet (Fig. 4e, f). Next, we fed HFD-mice with high or normal folate diet for 16 weeks, then harvested for analyses as below. High folate diet remarkably increased HFD-induced HCC development in *Mat2a* WT but not KO mice (Fig. 4g). Quantitative analyses revealed that high folate-feeding significantly increased the ratio of liver/body weight and maximum tumor size in liver in *Mat2a* WT but not KO mice (Fig. 4h, i). Furthermore, Ki67 staining demonstrates that high folate diet increased cell proliferation in *Mat2a* WT but not KO mice (Fig. 4j and Supplementary Fig. S5c). Meanwhile, we found that high folate diet led to profound elevation of the serum levels of AST and ALT in high fat-fed in *Mat2a* WT but not KO mice (Fig. 4k, l). Taken together, these results suggest that hepatic loss of *Mat2a* protects against HCC development, particularly folate-promoted HCC advancement.

### MATIIα and VCIP135 are important for HCC proliferation

**Fig. 5:**
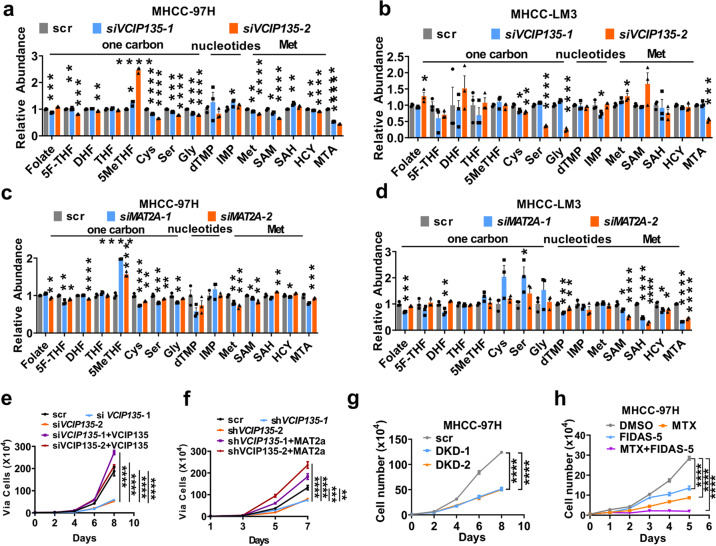
MATIIα and VCIP135 are essential for HCC proliferation. **a**, **b** Quantification of different metabolites of *VCIP135* knockdown in MHCC-97H cells (**a**) and MHCC-LM3 cells (**b**). Mean ± s.e.m. of *n* = 3 biologically independent experiments, one-way ANOVA test. **c**, **d** Quantification of different metabolites of *MAT2A* knockdown in MHCC-97H cells (**c**) and MHCC-LM3 cells (**d**). Mean ± s.e.m. of *n* = 3 biologically independent experiments, one-way ANOVA test. **e** *VCIP135* putback recovers *VCIP135* knockdown-induced reduction of cell proliferation in MHCC-LM3 cells. Mean ± s.e.m. of *n* = 3 biologically independent experiments, one-way ANOVA test. **f** *MAT2A* putback recovers *VCIP135* knockdown-induced reduction of cell proliferation in MHCC-LM3 cells in normal medium. Mean ± s.e.m. of *n* = 3 biologically independent experiments, one-way ANOVA test. **g** The effects of *MAT2A* and *VCIP135* double knockdown on proliferation in vitro. Mean ± s.e.m. of *n* = 3 biologically independent experiments, one-way ANOVA test. **h** MTX and FIDAS-5 synergistically repress cell proliferation. Mean ± s.d. of *n* = 3 biologically independent experiments, one-way ANOVA test. n.s. donates for no significance, *P < 0.05, **P < 0.01, ***P < 0.001 and ****P < 0.0001

Next, we detected the role of VCIP135 in folate metabolism and found that *VCIP135* knockdown significantly affected the abundance of intermediates of folate metabolism and methionine cycle, like SAM, SAH, and MTA in HCC cells (Fig. 5a, b). Cellular levels of most detected intermediates were downregulated by *VCIP135* knockdown while there was increased level of individual intermediates in different tested cell lines. Similar changing patterns of cellular intermediates were observed in HCC cells with *MAT2A* knockdown as well (Fig. 5c, d). The variation of HCC cell lines may contribute to the different changes of individual intermediates when silencing *VCIP135* or *MAT2A*.

We then investigated the functional role of VCIP135 and MAT2A in HCC cells growth. *MAT2A* knockdown suppressed cell proliferation and the rescue experiment showed cell proliferation was enhanced by *MAT2A* putback (Supplementary Fig. S6a). Further analysis revealed that putback of either *VCIP135* or *MAT2A* effectively restored cell proliferation curbed by silencing *VCIP135* (Fig. 5e, f and Supplementary Fig. S6b). In addition, double knockdown of *MATIIα* and *VCIP135* dramatically inhibits the growth in Huh7 and MHCC-97H cells (Fig. 5g and Supplementary Fig. S6c). Meanwhile, we found that MTX and FIDAS-5 (MAT2A inhibitor) synergistically repressed cell proliferation (Fig. 5h).

### MATIIα and VCIP135 are upregulated in HCC and significantly associated with poor prognosis

**Fig. 6:**
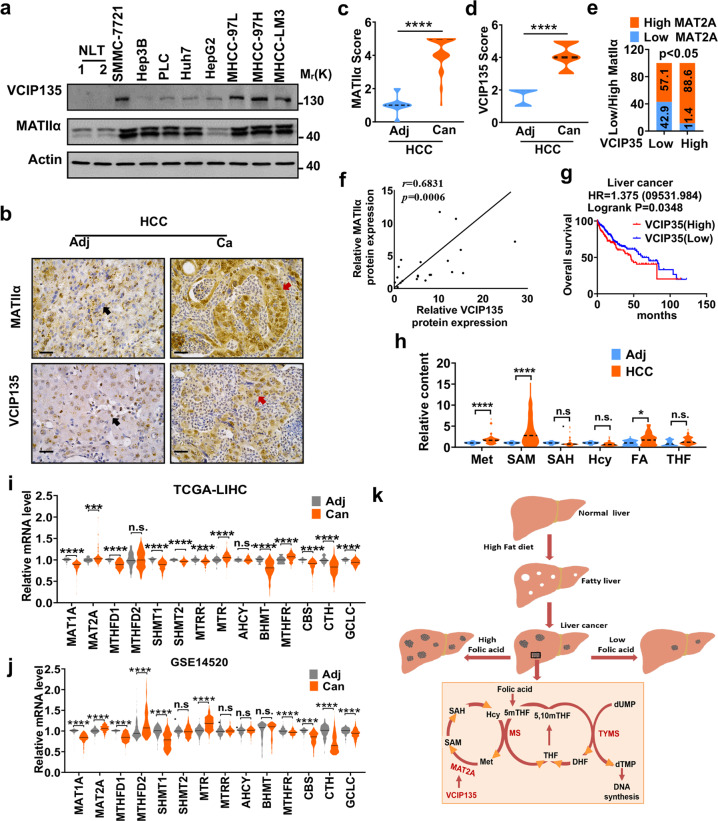
MATIIα and VCIP135 are upregulated in HCC and significantly associated with poor prognosis. **a** VCIP135 and MATIIα protein levels were detected by immunoblotting from HCC cell lines. **b** Represent images of IHC staining with antibodies against MATIIα or VCIP135 from human HCC patients (*n* = 58). **c**, **d** Quantification of MATIIα (**c**) and VCIP135 (**d**) protein levels according to IHC scores in HCC (*n* = 58). Adj, adjacent; Can, Cancer. Median indicated as dotted lines, two-tailed t-test. **e** Positive correlation of VCIP135 with MATIIα protein levels determined by IHC staining. The 58 samples were classified into two groups (High VCIP135, 51; Low VCIP135, 7) based on the VCIP135 level. **f** Positive correlation of VCIP135 with MATIIα protein levels in human hepatocellular cancer tissues samples. VCIP135 and MAT2A protein expression were detected by immunoblotting (*n* = 21). Spearman correlation, two-tailed. **g** Kaplan–Meier survival curves for patients with high VCIP135 expression have poorer overall survival compared with patients with low VCIP135 expression in liver cancer. Cutoff value used in analysis is 706. Log-rank (Mantel-Cox) test. FA, folic acid. **h** Levels of one-carbon metabolites from paired HCCs and non-tumor liver tissues of patients, (*n* = 44 per group). Median indicated as dotted lines, two-tailed t-test. **i**, **j** Different one-carbon metabolism-related genes expression levels in patients (TCGA, **i** and GSE14520, **j**). Median indicated as dotted lines, two-tailed t-test. **k** Working model. Higher folate diet enhances while folate free diet attenuates HCC development. Data in **a** is representative of three independent experiments. n.s. donates for no significance, *P < 0.05, **P < 0.01, ***P < 0.001 and ****P < 0.0001

Previous studies have demonstrated upregulation of MATIIα in HCC.^6,31^ To define the clinical relevance of MATIIα and VCIP135 in human HCC, we analyzed the expression of MATIIα and VCIP135 in HCC cell lines and found that both MATIIα and VCIP135 were upregulated in most HCC cell lines compared with normal liver tissues (Fig. 6a). Next, we examined the expression of MATIIα and VCIP135 in cancer samples and their matched adjacent tissues from HCC patients by immunoblotting. Remarkably, we detected higher MATIIα and VCIP135 protein levels in nearly all of the 21 HCC cancer samples than that in paired adjacent tissues (Supplementary Fig. S7a, b). Consistently, immunohistochemistry (IHC) analysis of the 58 paired HCC samples confirmed upregulation of MATIIα and VCIP135 expression in liver cancer tissues (Fig. 6b–d). We then analyzed the relationship between MATIIα and VCIP135 expression in tumor tissues. Immunoblotting and immunohistochemistry staining revealed that elevation of VCIP135 protein level was in line with increased MATIIα protein levels in HCC tissues (Fig. 6e). Spearman correlation analysis demonstrated the positive correlation between VCIP135 protein and MATIIα protein in human HCC (Fig. 6f). Moreover, we investigated whether overcome of patients with cancers could be predicted by *VCIP135* expression. Indeed, high *VCIP135* expression level was significantly associated with poor outcome of patient with liver cancer (Fig. 6g). We thus were curious to unravel the metabolic profile of folate and methionine metabolism in HCC patients and performed an unbiased HPLC-MS-based metabolic analysis of one-carbon metabolites in clinical tissues. Enrichment of methionine and SAM were observed in cancer tissues compared with that in adjacent tissues (Fig. 6h and Supplementary Fig. S7c). Intriguingly, many genes involved in one-carbon metabolism are upregulated in HCC according to TCGA and GEO data analyses (Fig. 6i, j). Collectively, these results demonstrate that MATIIα and VCIP135 coordinately control the folate effect on HCC development (Fig. 6k).

## Discussion

One of the prominent hallmarks of cancer is the remodeling of cellular metabolism that supports cancer cell proliferation and tumor growth. Cancer metabolism is characterized by abnormal alteration of metabolic demands, nutritional supplies, and dysregulation of metabolic enzymes activity compared to corresponding healthy tissue. An attracting aspect of cancer metabolism study is to define metabolic function of methionine, one of the essential amino acids. Its biological and therapeutic impact has been widely explored in the context of ageing and metabolic diseases, including cancer.^11,32–38^ Beyond influencing cell proliferation, increased metabolic flux derived from methionine cycle is critical to drive tumorigenesis caused by TICs.^12^ Dysregulated methionine metabolism in human hepatocellular cancer has also been reported.^31^ MATs are cellular enzyme that catalyze the formation of SAM from methionine and ATP. MATIIα, is a MAT isozyme expressed highly in fetal liver. However, its expression is decayed to negligible levels after birth and increased in the adult liver upon liver injury and fibrosis.^9,39,40^ Knocking down *MAT2A* gene expression causes cell cycle arrest and apoptosis in various cancer cells.^41,42^ In present study, liver-specific knockout of *Mat2a* remarkably abolishes HCC development in HFD-induced HCC mouse model. Of note, the effect of high or free of folate-diet on HFD-induced HCC relies on Mat2a. Besides, we also found that liver-specific knockout of Mat2a causes expression changes of various genes that involved in tumor development, although the underlying mechanism still remains unclear. Meanwhile, the switch expression of *MAT1A*/*MAT2A*, accompanied with a decrease of SAM acting on tumor suppressor, is occurred in HCC, which has been attributed to that catalytic activity of MAT1A is higher than that of MAT2A and SAM plays a function of tumor suppression.^31,43^ On the contrary, our study found that *MAT2A* knockdown decreased methionine cycle. SAM and MTA levels were downregulated in our tested condition. Interestingly, in line with our findings, elevated SAM and MTA denote for T cell exhaustion in HCC cancer,^44^ and *MAT2A* KO results in obstruction of T cell exhaustion and curbs tumor formation in mice by reducing SAM/MTA in liver.^44,45^ In addition, HCC induced by *glycine N-methyltransferase* (*GNMT*) KO in mice also displays increase of SAM and SAM-polyamine metabolic axis is critical to GNMT-null HCC.^46^ The discrepancies of the findings are probably due to differences of participated populations in clinical analysis or tumor heterogeneity. Indeed, emerging evidence reveals that HCC is highly heterogeneous.^47–49^

Folate is necessary for rapidly proliferating cells. Therefore, an increase of folate uptake is observed in different human solid cancers including ovarian, colorectal, kidney, and breast carcinomas.^50,51^ In addition, the chemotherapeutic drug, like methotrexate, raltitrexed, and edatrexate, that inhibits folate metabolism has been approved for cancer therapy for long period.^52–56^ These results suggest that folate plays an important role in cancer development. Indeed, in our study, we found that high folate-diet significantly promotes cancer development in HFD-induced HCC model. Meanwhile, high-folate feeding increases the protein level of MATIIα in cancer tissues. In contrast, folate-free diet reduces MATIIα expression and delays HFD-induced HCC development. Importantly, liver-specific knockout of *Mat2a* suppresses both the effect of high level or free of folate diet on HFD-induced HCC development. Accordingly, our metabolomic analyses showed that folate drives folate cycle and methionine cycle in HCC cells, revealing the essential role of folate-MATIIα metabolic axis in HCC development. It has been reported methionine is significantly increased in HCC compared to adjacent liver tissue.^57^ It is reasonable to speculate that elevated level of extracellular and/or intracellular folate initiates signaling transduction to increase methionine uptake and consumption by controlling hepatic MATIIα and causes methionine enrichment in liver during the proceeding of HCC development. It has been reported that the activity of MAT2A purified from rat liver is dramatically suppressed by SAM^58^ and the reduction of SAM in HCC were believed to release its inhibition on upregulated MAT2A. However, consistent with our current results, recent study found increased MAT2A, SAM, and other intermediates of methionine cycle, indicating metabolic plasticity in HCC.^44–46^ Interestingly, it has also been uncovered that the activity and stability of MATIIα proteins are regulated by post-translational modifications including phosphorylation, sumoylation, and acetylation. These modifications on MATIIα may also change its conformation to annul the inhibitory effect of SAM.^59–61^ Of note, recent discoveries of increase of MAT2A as well as elevation of SAM and other intermediates of methionine cycle, together with our current finding, indicate metabolic plasticity in HCC. And MAT2A activity may be regulated by different mechanisms based on the specific background of HCC cell lines, animal models, and populations of patients with HCC.

VCIP135 is an essential factor involved in the p97/p47 membrane fusion pathway and is required for Golgi biogenesis in vivo.^62^ Subsequently VCIP135 has been demonstrated to possess deubiquitylating activity. But the role of VCIP135 in cancer development still remains unclear. Here, we identified that VCIP135 binds to MATIIα to enhance MATIIα protein stability via deubiquitylation in response to folate treatment. MATIIα expression regulation has been widely studied. Both transcriptional and post-translational mechanisms are uncovered.^24^ Particularly, acetylating modification accelerates the degradation of MATIIα through ubiquitin-proteasome pathway.^7^ Although Cullin 3 has been demonstrated as E3 ligase of MATIIα in colorectal cancer cells, deubiquitylase of MATIIα, especially in HCC cells, is rare reported. Therefore, our study presents a novel target involved in MATIIα-associated metabolic disorders. Further functional experiments found that VCIP135 and MATIIα synergistically promote HCC proliferation in vitro.

More importantly, high expression levels of VCIP135 and MATIIα show significantly positive correlation in different cancers. By analyzing the data from the TCGA database, we demonstrated that upregulated expression of VCIP135 and MAT2A is also associated with poor prognosis of HCC patients, which is consistent with our current findings that MATIIα-integrated folate metabolism and methionine cycle promotes HCC development (Fig. 6k).

In summary, we unraveled the critical role of MATIIα in folate-promoted HCC development and underlying mechanism of the rewired methionine metabolism coupled with folate stress signal in HCC. Our findings provide a rational therapeutic strategy by targeting MATIIα and/or applying precisely dietary intervention with folate restriction to cancer patients displaying activated MATIIα metabolic pathway.

## Materials and methods

### Human subjects and data

Human hepatocellular carcinoma samples include primary tumors and adjacent non-tumor tissues that were surgically removed at the Fudan University Shanghai Cancer Center (Shanghai, China). All human materials were obtained with informed consent, and all procedures involving human samples followed the principles in the Declaration of Helsinki and approved by the Ethics Committee of Fudan University Shanghai Cancer Center (Shanghai, China). The TCGA cohorts data were downloaded from The Cancer Genome Atlas (https://cancergenome.nih.gov/) and/or UCSC Cancer Genomics Browser (https://xena.ucsc.deu/welcome-to-ucsc-xena/). Oncomine data were examined with the filters data type: mRNA, gene rank threshold: all; fold change threshold: 1.5; *p*-value threshold: 0.05 (https://www.oncomine.org/resource/login.html).

### Animals

Animal protocols were approved by the Institutional Animal Care and Use Committee at Fudan University. Animals were fed ad libitum and maintained in a specific pathogen-free facility with constant ambient temperature and a 12 h light cycle. Hepatocyte conditional *Mat2a* knockout (*Mat2a*^flox/flox^; *Alb-Cre*+) mice on the C57BL/6J background were generated by mating *Mat2a* ^flox/flox^ mice with Albumin-Cre mice. Hepatocellular carcinoma was induced by intraperitoneal (i.p.) with one dose of DEN (#N0725, Sigma Aldrich, St.Louis, MO) at 25 mg/kg in 2-week old mice. After 4 weeks, mice were either fed the high-fat diet (HFD, 60% fat in calories; Research Diets, #D12492, New Brunswick, NJ) or high-fat diet supplemented with standard folate (2 mg/Kg), high levels of folate (20 mg/Kg) or folate free as indicated for the desired periods until being sacrificed. The basic formulation of mice diet is referred to AIN-93M (adult maintenance) diet. We did not treat mice with succinylsulfathiazole to block folate production from gut microflora. Therefore, a severe side effect of folate deficiency could be prevented by gut microflora-derived folate. The mice had an impure genetic background.

### Cell culture studies

Cell lines were obtained from ATCC (HEK293T, HepG2, PLC/PRF/5, Hep3B), the Japanese Collection of Research Bioresources (Huh7), and Liver Cancer Institute, Fudan University (Shanghai, China) (MHCC-97L, MHCC-97H, MHCC-LM3). Only cell stocks that had tested negative for mycoplasma within the prior 9 months were used. All cells were cultured in DMEM with 10% FBS and 1% Pen Strep (GIBCO) and grown in a 37 °C humidified incubator with 5% CO_2_. For studies involving folic acid-free media, folic acid-free DMEM was prepared, and supplemented with purified folate (sigma) and/or 10% dialyzed FBS and 1% Pen Strep (GIBCO).

### Plasmid constructs

Flag-MAT2A, Myc-VCIP135 (WT), Myc-VCIP135 (C218S), HA-Ub were generated by cloning the coding region of human into pcDNA3.1 respectively. Oligos for shRNA were as follows: si*MAT2A*-UTR-1 GCUUGCUAUUCUGUCCCUATT; si*MAT2A*-UTR-2: CCUUGUGAUGUGCACGUAATT; si*MAT2A*-CDS-3: GGAUCGAGGUGCUGUGCUUTT. sh*VCIP135*-4: GGACAAACATGGTATCCATCC; sh*VCIP135*-8: GAAAGTTGTCCACACTATATT.

### Immunoprecipitation and western blotting

Cells were lysed in 0.3% Nonidet P40 buffer (150 mM NaCl, 50 mM Tris-HCl, pH 7.5) containing inhibitors (1 mM phenylmethylsulphonyl fluoride, 1 μg/ml of aprotinin, 1 μg/ml of leupeptin, 1 μg/ml of pepstatin, 1 mM Na_3_VO_4_, 1 mM NaF, all in their final concentrations). The lysates were pre-cleared and incubated with the indicated primary antibodies and Protein G agarose (11243233001, Roche) or incubated with anti-Flag M2 agarose (Sigma). Each protein sample has been extracted from human liver samples or cultured cells were subjected to SDS/PAGE and transferred to NC membrane (GE Health). Then the corresponding primary and secondary antibodies were incubated to visualize the protein.

Information of primary antibodies used for WB are as follows: MAT2A, Genetex, GTX50027, 1:2000; MAT1A, abcam, ab229609, 1:2000; VCIP135, CST, 88153S, 1:1000; HA, SAB, T501, 1:3000; Flag, Aogma, #9622, 1:10000; β-actin, Aogma, #9601, 1:10,000; Myc, CST, 2276s, 1:1000. Information of primary antibody used for IP is as follows: VCIP135, CST, 88153S, 1:100.

### RNA-seq and data analysis

Total RNA was extracted from liver tissues in biological triplicates from 4 weeks *Mat2a* WT and LKO mice by using illustra RNAspin Mini Kit (GE Healthcare). RNA samples were quantified using Nanodrop and qualified by agarose gel electrophoresis. Illumina kits which include procedures of RNA fragmentation, random hexamer primed first strand cDNA synthesis, dUTP-based second strand cDNA synthesis, end-repairing, A-tailing, adaptor ligation, and library PCR amplification, were used for RNA-seq library preparation. Finally, the prepared RNA-seq libraries were qualified using Agilent 2000 Bioanalyzer and quantified by qPCR absolute quantification method. The sequencing was performed using Illumina HiSeq 4000.

Raw sequencing data generated from Illumina HiSeq 4000 that pass the Illumina chastity filter were used for the following analysis. Trimmed reads (trimmed 5′,3′-adaptor bases) were aligned to reference genome. Base on alignment statistical analysis (mapping ratio, rRNA/mRNA content, fragment sequence bias), we determine whether the results could be used for subsequent data analysis. If so, the expression profiling, differentially expressed genes, and differentially expressed transcripts were calculated. The novel genes and transcripts were also predicted. Principal Component (PCA), Correlation Analysis, Hierarchical Clustering, Gene Ontology (Go), Pathway Analysis, scatterplots, and volcano plots were performed for the differentially expressed genes in R or Python environment for statistical computing and graphics.

### Quantitative reverse-transcription PCR

RNA was extracted from liver tissue using Trizol reagent (Invitrogen) according to the manufacturer’s protocol. The cDNA was synthesized from purified RNA using the PrimeScript RT reagent Kit (Takara). Quantitative PCR was performed using the Step One Plus Real-Time PCR System (Applied Biosystems) in a 10 μl reaction mixture containing cDNA, SYBR Green Master Mix (Takara), and mouse-specific oligonucleotides. The MRPL13A gene was used as internal control. The data were expressed as a relative fold-change in comparison to the control.

### In vitro ubiquitylation assays

The cells were lysed with 1% SDS lysis buffer (50 mM Tris-HCl, pH 7.5, 0.5 mM EDTA, 1 mM dithiothreitol) with inhibitors and boiled for 10 min. After centrifugation, the supernatants were subjected to immunoprecipitation and western blot analysis with specific antibodies.

### Histological and immunohistochemically analysis

Sectioned liver tissues were fixed in 10% formalin and embedded in paraffin. Sections were cut at 7 μm, deparaffinized, subjected to citrate buffer antigen retrieval, and exposed to hydrogen peroxide to quench endogenous peroxidase prior to incubation with primary antibodies. Vectastain ABC kit and ImmPACT DAB (Vector Laboratories) were used for chromogen development, followed by counterstaining with Harris Hematoxylin. Information of primary antibodies used for IHC is as follows: MAT2A, Atlas antibodies, HPA043028, 1:200; MAT1A, abcam, ab229609, 1:400; VCIP135, Novus, NBP1-49939, 1:100; Ki67, abcam, ab15580, 1:1000.

IHC staining was scored as previously described.^63^ Briefly, score is based on the percentage of positive-staining area (0, 1 = 25%, 2 = 50%, 3 = 75% and 4 = 100%) and staining intensity (none, low, medium and high, graded as 0, 1, 2 and 3, respectively). The percentage of positive-staining area in each field was counted as positive area/total area ×100%. Statistical methods were indicated in each figure legend.

#### Tumor incidence calculation

Liver tissues from experimental mice were collected and subjected to HE staining to identify the occurrence of tumors. Tumor incidence of each group represents numbers of mice bearing tumors/total numbers of mice assigned in each group.

### Cell proliferation assays

Cells were plated in triplicate in six-well plates at 2000 to 10,000 cells per well. The culture medium was supplemented with 10% FBS, 25 mM glucose. Cell numbers were counted with trypan blue staining to determine cell viability.

#### TAP-MS

The control plasmid and flag-tagged experimental plasmid were transfected into HEK293T cells. 48 h later, the cells were collected and washed twice with cold PBS. 0.3% NP-40 (150 mM NaCl, 50 mM Tris, 3 ml NP-40, pH7.5) and protease inhibitors were added and the solution was incubated on ice for 30 min. Then supernatants were collected by centrifugation at 4 °C and 120,00 × *g* for 20 min and incubated with Flag beads at 4 °C for 4 h. Then the beads were washed with 0.3%NP-40 for three times. 60 µ 1 1 X loading buffer was added and taken metal bath at 100 °C for 10 min. The sample was subjected to SDS-PAGE, following by the gel cut off for mass spectrometry. The score was calculated by *mascot* software and represents the credibility of results.

#### Metabolites analysis and liquid chromatography-mass spectrometry analysis (LC-MS)

For liver tissue, 100 mg samples were collected and homogenate (60 Hz, 90 s) in 0.8 ml of ice-cold 80% methanol and 20% ddH2O. For culture cells, 1 × 10^7^ cells were collected and resuspended in 0.8 ml of ice-cold 80% methanol and 20% ddH_2_O. Then the samples were vigorously vortexed and placed in liquid N_2_ for 10 min to freeze, then thawed on ice for 10 min. The freeze–thaw cycle was repeated twice. Samples were centrifuged at 13,000 × *g* for 15 min and the supernatant was collected for analysis. Metabolites were normalized on the basis of protein concentration.

#### Glucose tolerance test (GTT) and insulin tolerance test (ITT)

For GTT assay, mice were fasted overnight and 2 g/kg glucose was injected *via* enterocoelia. The concentration of blood glucose was measured at the indicated time points after injection. For ITT assay, mice were fasted for 6 h during day time and blood samples were collected from the tail vein. Blood glucose concentration was detected at the defined time points after injection of 0.75 U/kg insulin *via* enterocoelia.

### Statistical analysis

All data in this study were presented as the mean ± s.d from one representative experiment of multiple independent experiments. Statistical analysis was conducted using unpaired two-tailed t-test, one-way or two-way analysis of variance (ANOVA), followed by Fisher’s least significant difference (assuming equal variances) with SPSS software (version 21.0). Spearman correlation analysis was conducted by Graphpad Prism. *P*-values less than 0.05 were considered statistically significant.

## Supplementary information

figure S1-S7

## Data Availability

The RNA-seq data reported in this paper have been deposited in the Genome Sequence Archive in National Genomics Data Center, China National Center for Bioinformation/Beijing Institute of Genomics, Chinese Academy of Sci-ences (GSA: CRA008805) that are publicly accessible at https://ngdc.cncb.ac.cn/gsa. All the other data shown in this paper are available from the corresponding authors upon reasonable request.
