# Correction To: Dietary folate drives methionine metabolism to promote cancer development by stabilizing MAT IIA

**DOI:** 10.1038/s41392-022-01255-w

**Published:** 2022-12-28

**Authors:** Jin-Tao Li, Hai Yang, Ming-Zhu Lei, Wei-Ping Zhu, Ying Su, Kai-Yue Li, Wen-Ying Zhu, Jian Wang, Lei Zhang, Jia Qu, Lei Lv, Hao-Jie Lu, Zheng-Jun Chen, Lu Wang, Miao Yin, Qun-Ying Lei

**Affiliations:** 1 Fudan University Shanghai Cancer Center & Institutes of Biomedical Sciences; Cancer Institutes; Key Laboratory of Breast Cancer in Shanghai; Shanghai Key Laboratory of Radiation Oncology, the Shanghai Key Laboratory of Medical Epigenetics, Department of Oncology, Shanghai Medical College, Fudan University, Shanghai, 200032 People’s Republic of China; 2 Department of Hepatic Surgery, Fudan University Shanghai Cancer Center, Shanghai Medical College, Fudan University, Shanghai, 200032 People’s Republic of China; 3 MOE Key Laboratory of Metabolism and Molecular Medicine, Department of Biochemistry and Molecular Biology, School of Basic Medical Sciences, Fudan University, Shanghai, 200032 People’s Republic of China; 4 State Key Laboratory of Cell Biology, Shanghai Institute of Biochemistry and Cell Biology, Center for Excellence in Molecular Cell Science, Chinese Academy of Sciences (CAS), School of Life Science and Technology, Shanghai Tech University, Shanghai, 200032 People’s Republic of China; 5 State Key Laboratory of Medical Neurobiology, Fudan University, Shanghai, 200032 People’s Republic of China

**Keywords:** Cancer metabolism, Gastrointestinal cancer

Correction to: *Signal Transduction and Targeted Therapy* 10.1038/s41392-022-01017-8, published online 22 June 2022

In the process of collating the raw data, the authors noticed one inadvertent mistake occurred in Fig. 2e that needs to be corrected in the article^1^. The correct data are provided as follows. The key findings of the article are not affected by the correction. The original article has been corrected.
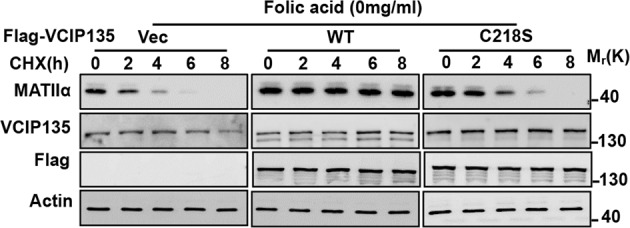


Fig. 2e VCIP135 WT but not enzymatic-dead mutant stabilizes MATIIα upon folate deprivation for 72 h. MHCC-97H cells were cultured under folate-deprived condition, followed by CHX treatment.

In addition, the RNA-seq data deposited in Genome Sequence Archive (GSA) database and shared. Thus, in “DATA AVAILABILITY” part is changed, to “The RNA-seq data reported in this paper have been deposited in the Genome Sequence Archive in National Genomics Data Center, China National Center for Bioinformation / Beijing Institute of Genomics, Chinese Academy of Sci-ences (GSA: CRA008805) that are publicly accessible at https://ngdc.cncb.ac.cn/gsa. All the other data shown in this paper are available from the corresponding authors upon reasonable request”.

## References

[1] Li JT (2022). Dietary folate drives methionine metabolism to promote cancer development by stabilizing MAT IIA. Signal Transduct Target Ther.

