# Supplementary material for: Dietary folate drives methionine metabolism to promote cancer development by stabilizing MAT IIA
Source: Signal Transduct Target Ther. 2022 Jun 22;7:192. doi: 10.1038/s41392-022-01017-8 (PMC9213445; doi:10.1038/s41392-022-01017-8)
Supplement: Supplementary file 1 — figure S1-S7 [file 41392_2022_1017_MOESM1_ESM.docx]

Supplementary Materials for

Dietary folate drives methionine metabolism to promote cancer development by stabilizing MATIIA

Jin-Tao Li^1#^, Hai Yang^1#^, Ming-Zhu Lei^1#^, Wei-Ping Zhu^2#^, Ying Su^1^, Kai-Yue Li^1^, Wen-Ying Zhu^1^, Jian Wang^1^, Lei Zhang^1^, Jia Qu^1^, Lei Lv^3^, Hao-Jie Lu^1^, Zheng-Jun Chen^4^, Lu Wang^2*^, Miao Yin^1*^, Qun-Ying Lei^1,5,6*^

*Correspondence to: qlei@fudan.edu.cn, [miaoyin@fudan.edu.cn](mailto:miaoyin@fudan.edu.cn) or

wanglushanghaicancercenter@hotmail.com

**This PDF file include:**

Figures. S1 to S7

**Figure. S1.**


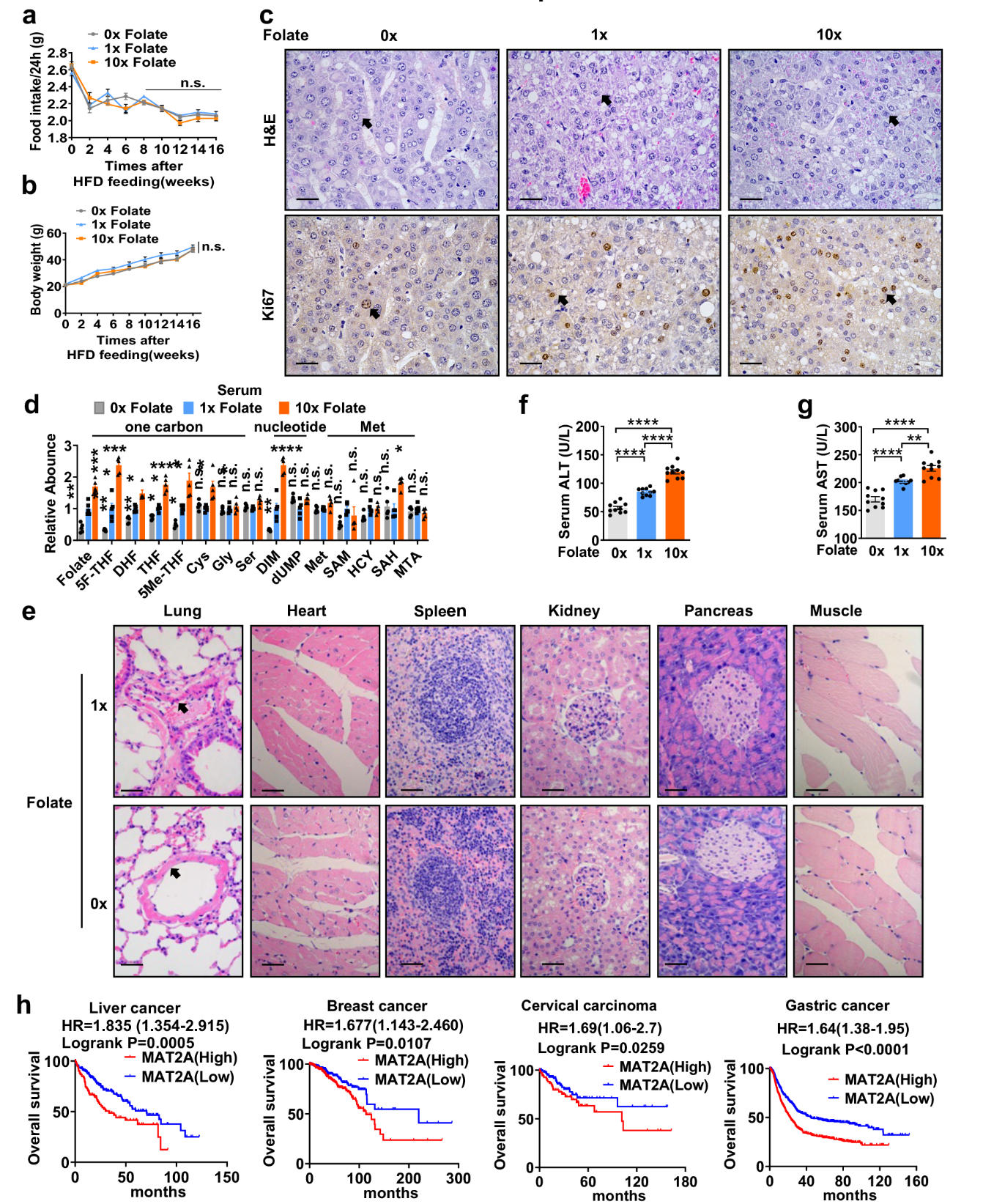


**Supplementary Fig. 1 MATIIα positively correlates with folic acid-promoted cancer development. a**,**b** Food intake and body weight in mice fed with different folate diet. Mean ± s.e.m. of n = 4 (**a**) and 10 (**b**) biologically independent experiments, one-way ANOVA test. **c** Liver sections obtained from indicated mice (n = 9 - 10) were stained with H&E or immunohistochemical stained with antibodies against Ki67. **d** Quantification of different metabolites of liver tissue in mice fed with different folate diets. Mean ± s.e.m. of n = 5 biologically independent experiments, one-way ANOVA test. **e** Representative images of H&E staining in different organs in mice fed with different folate diets. **f**,**g** ALT (**f**) and AST (**g**) contents in mice serum. Mean ± s.e.m. of n = 10 biologically independent experiments, one-way ANOVA test. **h** Kaplan-Meier survival curves demonstration of overall survival of patients with high/low *MAT2A* expression in multiple tumor types. Cutoff value used in analysis is 2267, 4230, 4414, 2718 in liver, breast, cervical and gastric cancer, respectively. Log-rank (Mantel-Cox) test. Scale bars: 12.5 μm. n.s. donates for no significance, *P < 0.05, **P < 0.01, ***P < 0.001 and****P < 0.0001.

**Figure. S2.**


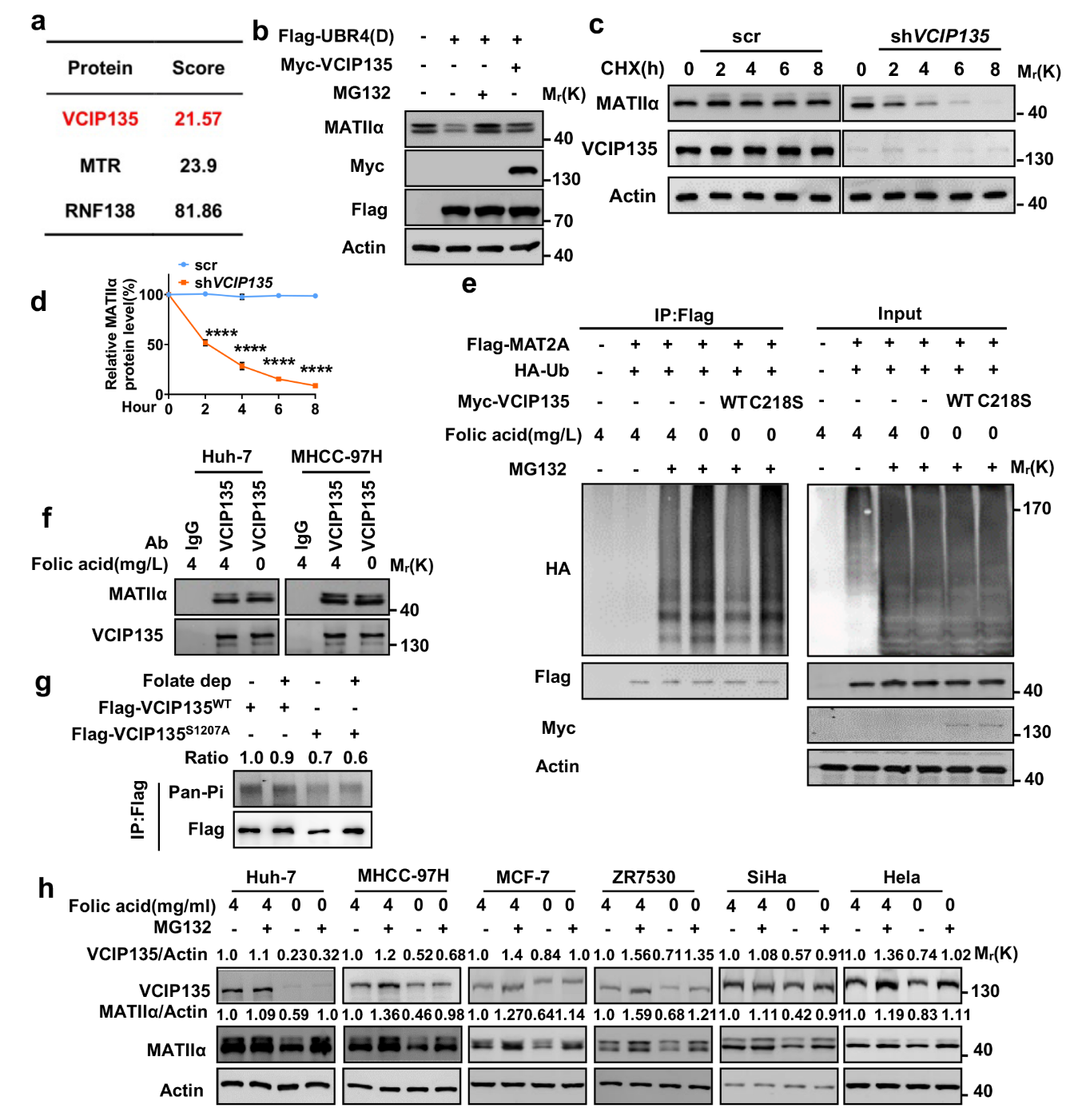


**Supplementary Fig. 2 DUB VCIP135 senses folic acid to stabilize MATIIα. a** Identification of interaction proteins with MATIIα by TAP-MS. Only VCIP135 is a DUB. **b** Overexpressed VCIP135 rescues E3 ubiquitin ligase UBR4-mediated MATIIα degradation. HEK293T cells were transfected with the indicated plasmids. **c**,**d** *VCIP135* knockdown destabilizes MATIIα. *VCIP135* WT or knockdown cells were treated with CHX as indicated and cell lysates directly subjected to immunoblots. Mean ± s.d. of n = 3 biologically independent experiments, two-tailed t-test. **e** VCIP135 WT but not enzymatic-dead mutant blocks folate deprivation-induced ubiquitylation of MATIIα. HEK293T cells were transfected as indicated and cultured in presence or absence of folate. Ubiquitylation assay was conducted. **f** Folic acid does not affect the interaction between VCIP135 and MATIIα. MHCC-97H and Huh7 cells were cultured with or without folate for 48h and treated with MG132 for 6h before harvest. Interaction between endogenous VCIP135 and MATIIα was determined by Co-IP and immunoblots. **g** Folate deprivation has no effect on VCIP135 activity. **h** VCIP135 and MATIIα protein level is decreased under folate deprivation for 72 hours in multiple cancer cells. Data (**b** and **c**, **e**-**g**) are representative of 3 independent experiments. ****P < 0.0001.

**Figure. S3.**

**D**


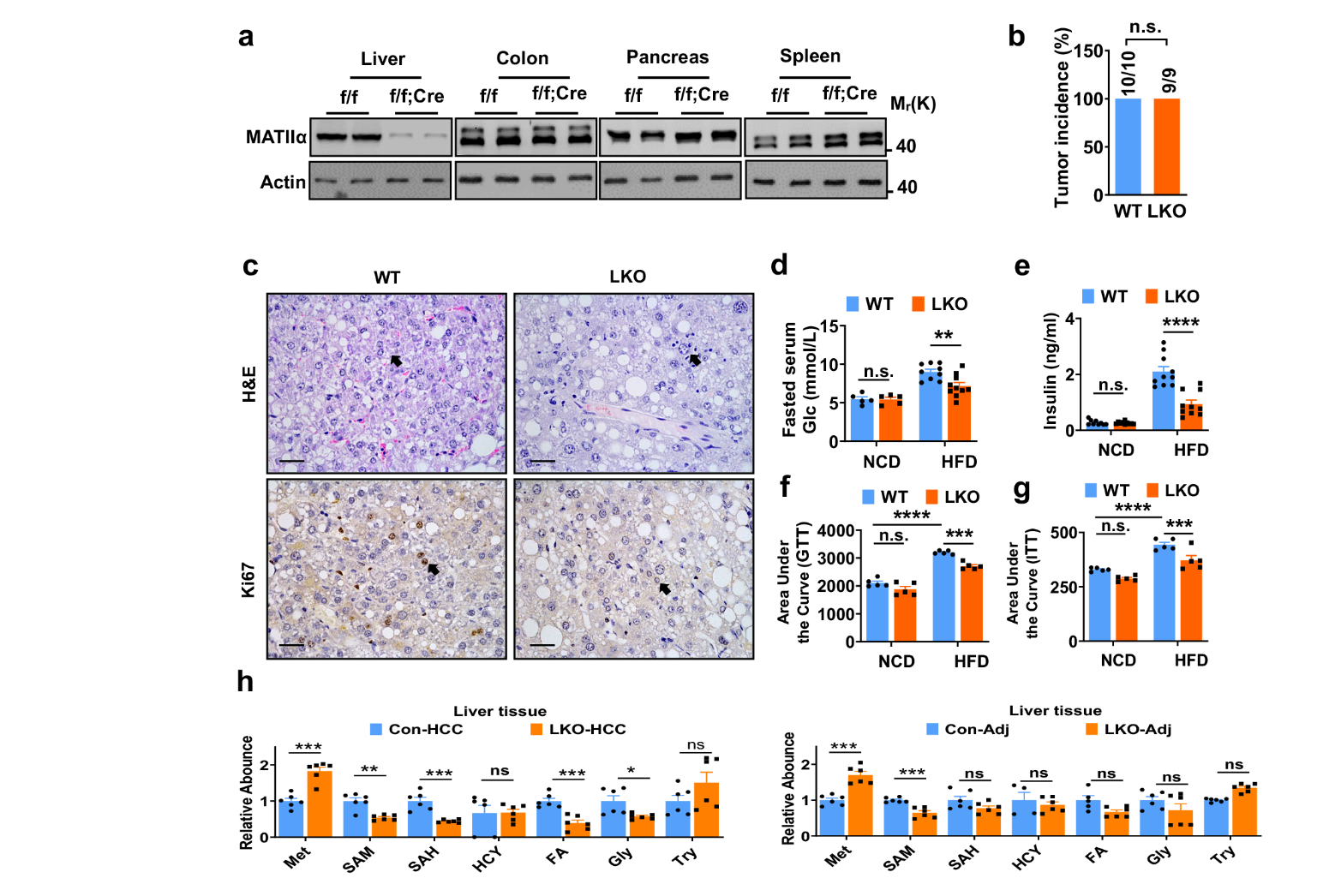


**Supplementary Fig. 3 MATIIα mediates DEN/HFD induced HCC development. a** Identification of liver tissue-specific *Mat2a*-knockout mice. **b** Quantification of the incidence of tumors induced by DEN/HFD in indicated mice after HFD induced *Mat2a* LKO mice. Mean ± s.e.m., two-tailed t-test. **c** Liver sections obtained from indicated mice (n = 9-10) were stained with H&E or immunohistochemical stained with antibodies against Ki67. **d**,**e** Quantification of fasted serum glucose and insulin in HFD induced *Mat2a* LKO mice. Mean ± s.e.m. of n = 5 -10 biologically independent experiments, two-tailed t-test. **f**,**g** Blood glucose levels after NCD or HFD feeding for 4 months in *Mat2a* WT or LKO mice during GTT and ITT. Mean ± s.e.m. of n = 5 biologically independent experiments, two-tailed t-test. **h** Metabolites detection in *Mat2a*-KO mice model. Mean ± s.e.m. of n = 6 biologically independent experiments, one-way ANOVA test. Data in **a** is representative of 3 independent experiments. Scale bars, 12.5 μm. n.s. donates for no significance, **P < 0.01, ***P < 0.001 and****P < 0.0001.**Figure. S4.**


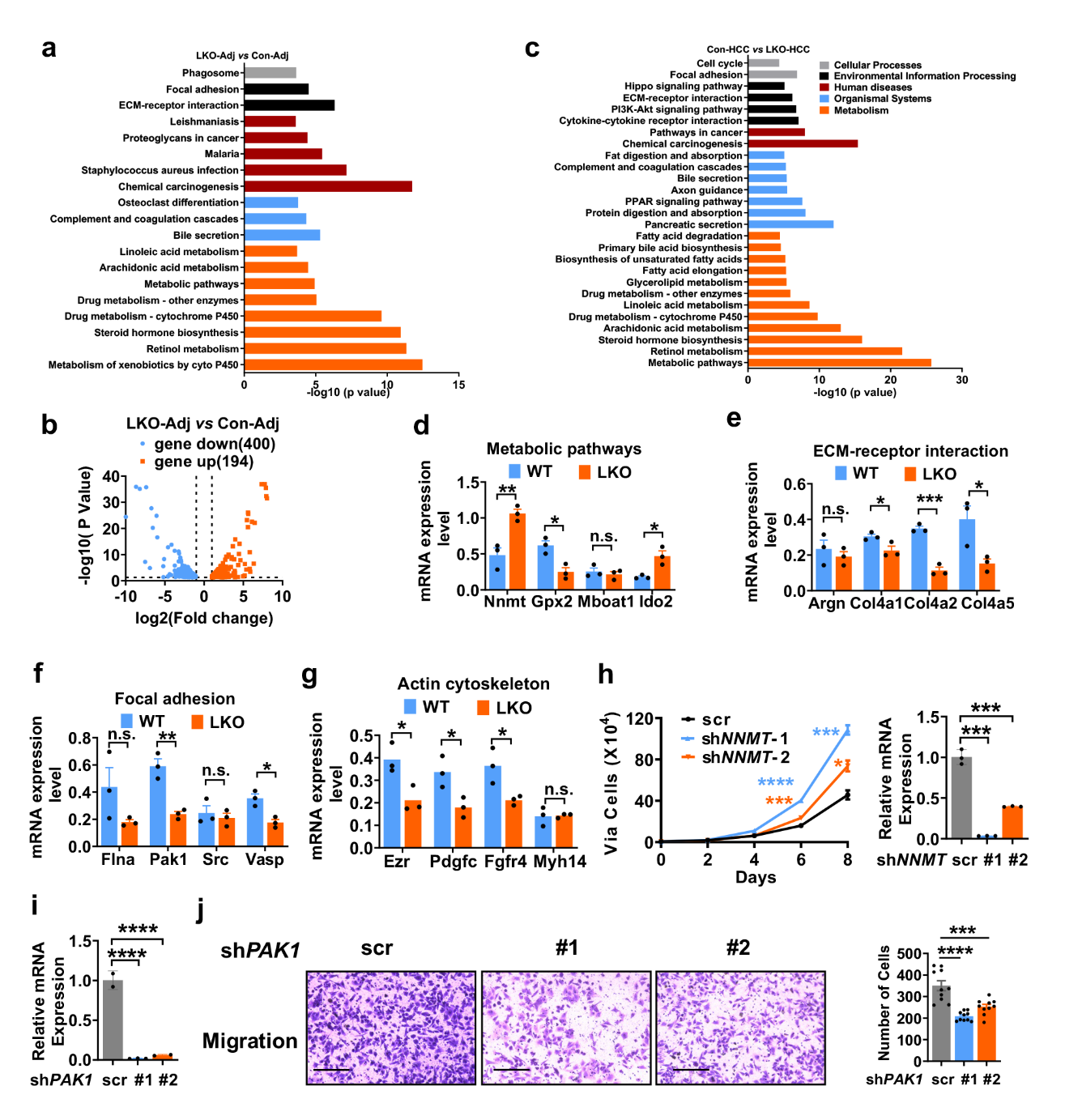


**Supplementary Fig. 4 Gene expression profile of *Mat2a* knockout mice. a,b** KEGG and Volcano map shows the altered genes and pathways in mice liver via RNA-seq. **c** KEGG analysis show the altered genes and pathways in mice liver *via* RNA-seq after *Mat2a* specific knockout in the hepatocytes with 16-week DEN/HFD treatment. **d**-**g** Relative expression of genes encoding for metabolism pathways (**d**) ECM-receptor interaction (**e**) Focal adhesion (**f**) and Actin cytoskeleton (**g**) *via* Q-PCR in mice liver with 32-week DEN/HFD treatment. Mean ± s.e.m. of n = 3 biologically independent experiments, two-tailed t-test. **h** *NNMT* knockdown increases cell proliferation. Mean ± s.e.m. of n = 3 biologically independent experiments, one-way ANOVA test. **i**,**j** *PAK1* knockdown decreases cell migration. Mean ± s.e.m. of n = 10 biologically independent experiments, one-way ANOVA test. Scale bar: 200 μm. n.s. donates for no significance, *P < 0.05, **P < 0.01, ***P < 0.001 and****P < 0.0001.

**Figure. S5.**


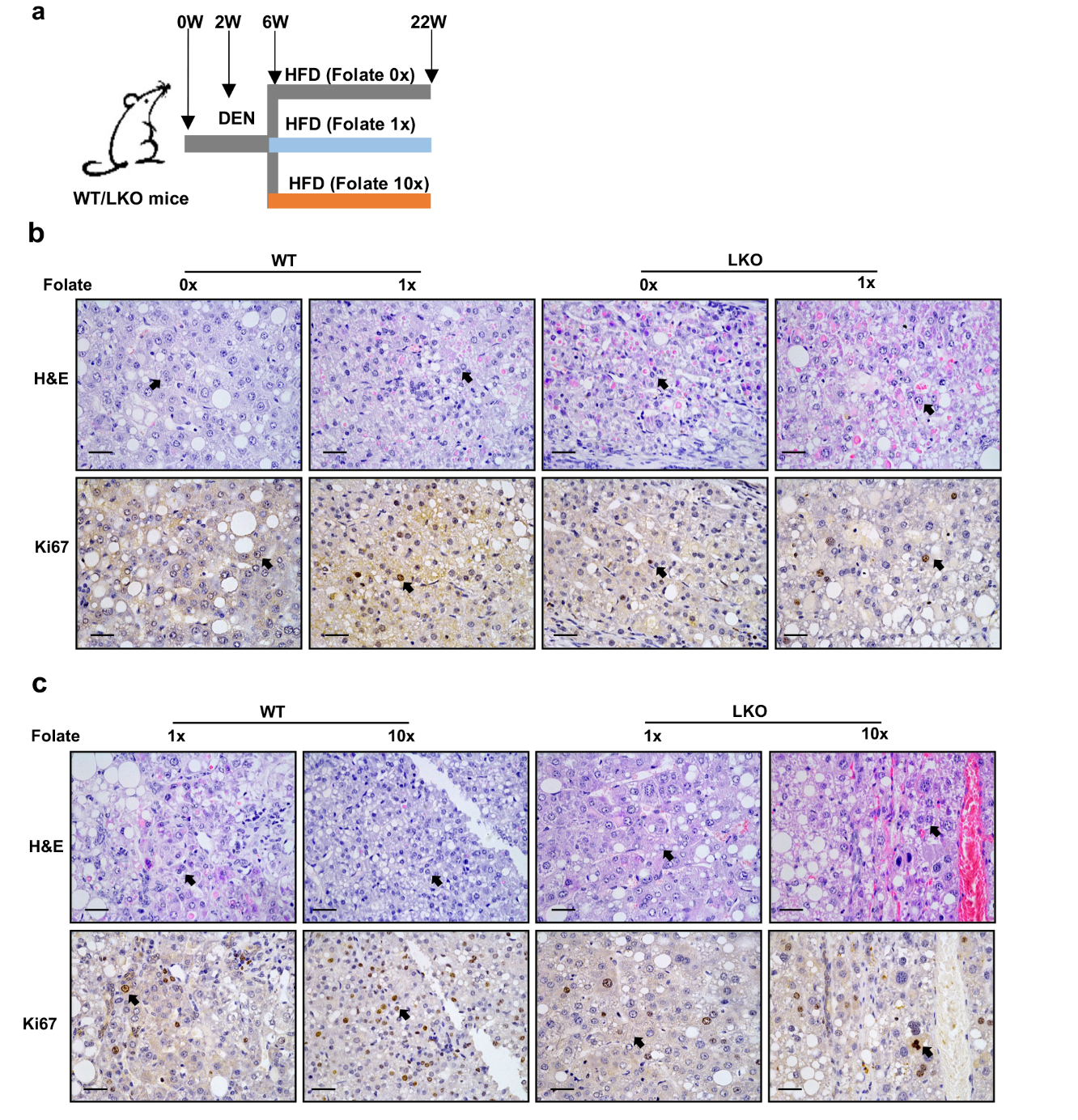


**Supplementary Fig. 5 MATIIα is essential for folic acid-promoted cancer development. a** Schematic representation of DEN/HFD-induced HCC model fed with different folate diet. Male C57BL/6J mice for WT group. **b** Liver sections obtained from indicated mice were stained with H&E or immunohistochemical stained with antibodies against Ki67. **c** Liver sections obtained from indicated mice were stained with H&E or immunohistochemical stained with antibodies against Ki67. Scale bars, 50μm.

**Figure. S6.**


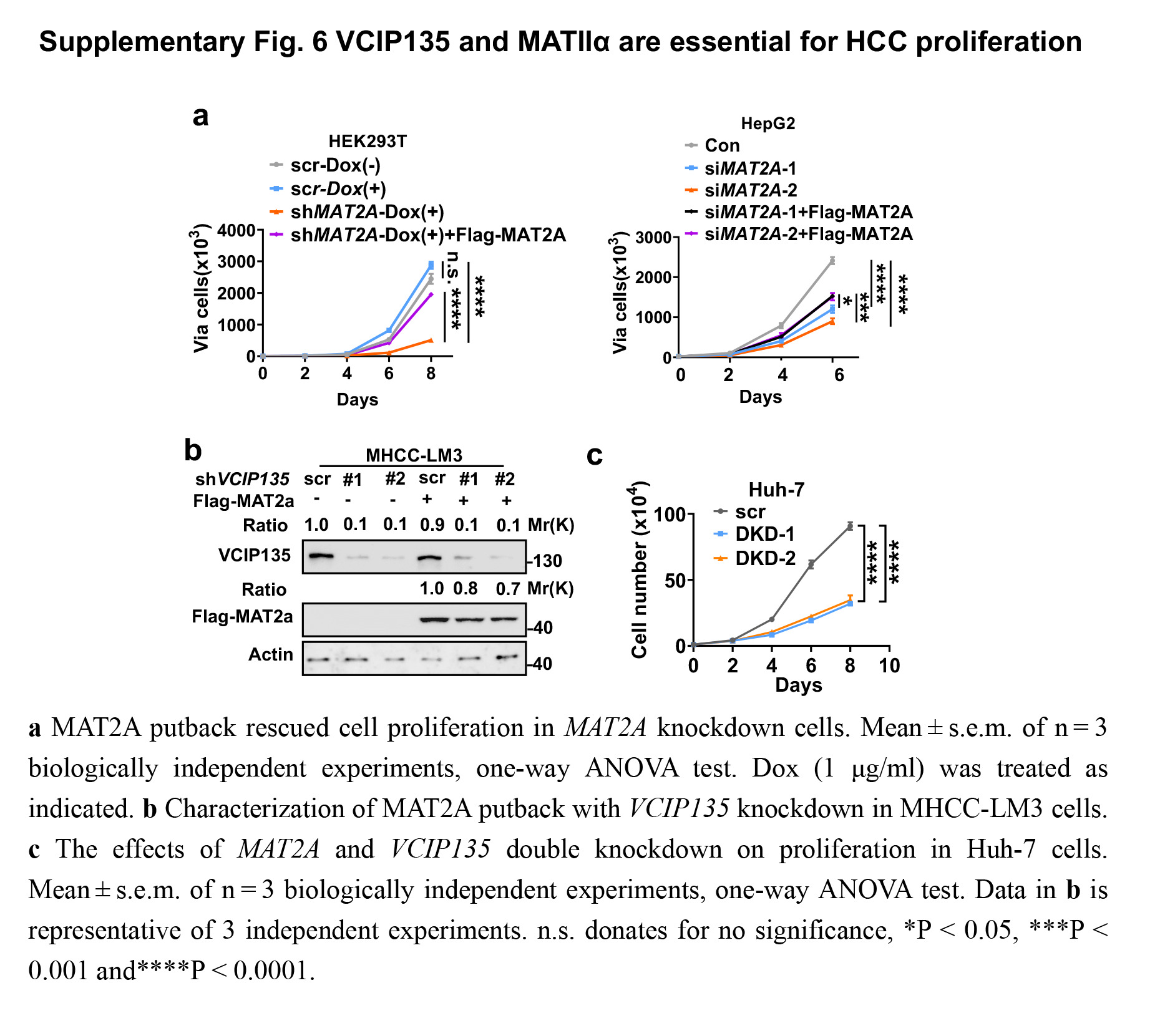


**Supplementary Fig. 6 VCIP135 and MATIIα are essential for HCC proliferation. a** MAT2A putback rescued cell proliferation in *MAT2A* knockdown cells. Mean ± s.e.m. of n = 3 biologically independent experiments, one-way ANOVA test. Dox (1 μg/ml) was treated as indicated. **b** Characterization of MAT2A putback with *VCIP135* knockdown in MHCC-LM3 cells. **c** The effects of *MAT2A* and *VCIP135* double knockdown on proliferation in Huh-7 cells. Mean ± s.e.m. of n = 3 biologically independent experiments, one-way ANOVA test. Data in **b** is representative of 3 independent experiments. n.s. donates for no significance, *P < 0.05, ***P < 0.001 and****P < 0.0001.

**Figure. S7.**


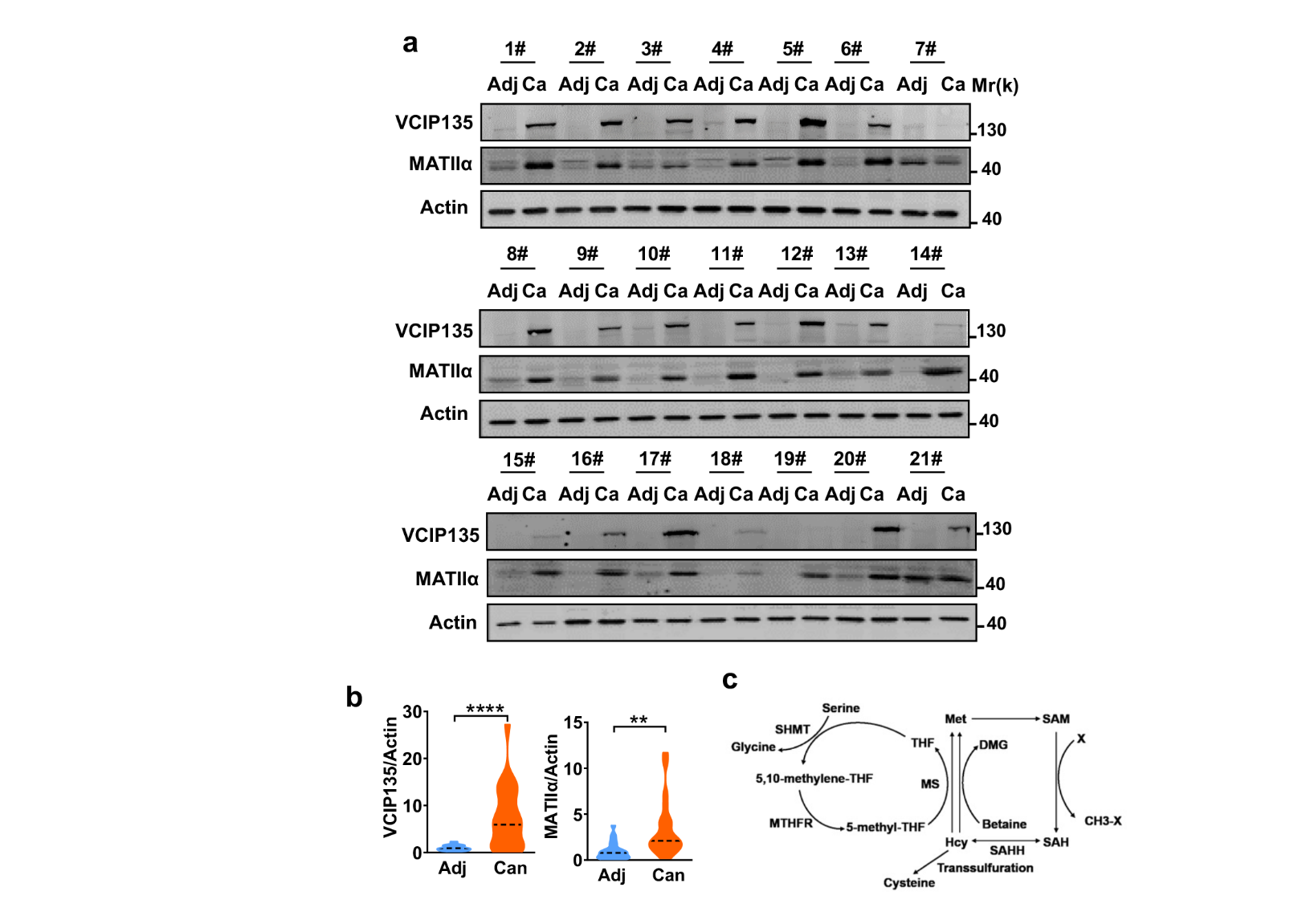


**Supplementary Fig.7 MAT2A and VCIP135 are positively correlated in HCC. a**,**b** Detection (**a**) and quantification (**b**) of VCIP135 and MATIIα protein levels by immunoblotting from paired human HCC samples (n = 21). The expression levels of MATIIα and VCIP135 protein were normalized to β-actin. Median indicated as dotted lines, two-tailed t-test. **c** Schematic representation of one-carbon metabolism. Data in **a** is representative of 3 independent experiments. Adjacent area and turmor area indicated as black arrow and red arrow, respectively. Scale bar: 12.5 μm. **P < 0.01 and****P < 0.0001.

**c**

**d**
